# HPV-Negative Adenocarcinomas of the Uterine Cervix: From Molecular Characterization to Clinical Implications

**DOI:** 10.3390/ijms232315022

**Published:** 2022-11-30

**Authors:** Luca Giannella, Jacopo Di Giuseppe, Giovanni Delli Carpini, Camilla Grelloni, Mariasole Fichera, Gianmarco Sartini, Serena Caimmi, Leonardo Natalini, Andrea Ciavattini

**Affiliations:** Woman’s Health Sciences Department, Gynecologic Section, Polytechnic University of Marche, 60123 Ancona, Italy

**Keywords:** adenocarcinoma, cervix, HPV, HPV-negative, therapy, molecular basis, gene mutations

## Abstract

Cervical cancer is the fourth most common cancer in women. It is the leading cause of female deaths in developing countries. Most of these cervical neoplasms are represented by squamous lesions. Cervical adenocarcinoma causes about a quarter of cervical cancers. In contrast to squamous lesions, cervical glandular disease is HPV-negative in about 15–20% of cases. HPV-negative cervical adenocarcinomas typically present in advanced stages at clinical evaluation, resulting in a poorer prognosis. The overall and disease-free survival of glandular lesions is lower than that of squamous lesions. Treatment options require definitive treatments, as fertility-sparing is not recommended. Moreover, the impact of HPV vaccination and primary HPV screening is likely to affect these lesions less; hence, the interest in this challenging topic for clinical practice. An updated review focusing on clinical and molecular characterization, prognostic factors, and therapeutic options may be helpful for properly managing such cervical lesions.

## 1. Introduction

The WHO estimates cervical cancer is the fourth most frequent cancer in women. The incidence was 570,000 cases in 2018, representing 6.6% of all female cancers worldwide [1,2,3].

SCC is the most common subtype of cervical cancer, and most histological groups are linked to HPV infection [4,5,6]. Adenocarcinomas comprise approximately 25% of cervical cancers, and they are much more heterogeneous than SCC, with 15% of them approximately unrelated to HPV infection [7,8]. Therefore, it has become evident that HPV does not drive all ECA. Furthermore, while there is a reduction in cervical squamous lesions, glandular lesions appear to increase [2]. In North America, cervical squamous carcinomas decreased from 95% to 67–84% over the past 20 years. In contrast, cervical adenocarcinomas increased from 5% to 8–27% [9].

As demonstrated for other organs, HPV status can significantly affect prognosis, and the different subtypes of ECA have different molecular patterns that could have significant clinical consequences for the survival of women [4,5,10]. In the era of HPV-vaccine and HP-only screening, it seems critical for clinical practice to distinguish ECA related to HPV infection from those not [2,3,11]. Currently, the study of NHPVAs represents a trending topic, given their different clinical course with worse outcomes in the face of guidelines that do not differentiate the management of these lesions from their squamous counterpart [2,3].

Therefore, a comprehensive review of this topic, including clinical, molecular, and prognostic characterization, may interest clinicians who deal with this pathology in their clinical practice.

## 2. Critical Issues in Diagnosing Non-HPV-Related Cervical Lesions

### HPV-Negative Cervical Cancer

There is no clear definition of HPV-negative cervical cancer. Yoshida et al. defined an HPV-negative cervical cancer as primary cancer diagnosed through its histological features, with a negative HPV test without a possible justification [4]. When dealing with HPV-test-negative cervical cancer, the clinician must consider three plausible reasons for the negative HPV result:

1. Real HPV-negative cervical cancer;
2. False HPV-negative case;
3. Incorrect classification of a non-cervical cancer.

**a.**  **Real HPV-negative cervical cancer**

A meta-analysis investigating 30,848 invasive cervical cancers between 1990 and 2010 found a gradual decrease in HPV negativity. The increasing trend of HPV-positive diagnoses (from 85.9% in 1990 to 92.9% in 2010) seems to correlate with an improvement in the sensitivity of HPV tests and a more accurate classification of histotypes [12]. 

Patients’ age influence the HPV infection rate in cervical carcinomas, material storage time, geographic factors, and, more than anything else, the histological subtype [13]. 

In 2017, a clinical study of the Cancer Genome Atlas estimated the prevalence of HPV in cervical carcinoma specimens collected in the United States: 5% resulted negative for HPV infection [14]. The overall incidence of HPV-negative cancer, reported by recent literature, fluctuates between 7 and 11% [4,14,15,16,17,18].

Several studies detected the presence of HPV in nearly 100% of cervical squamous cell carcinoma, confirming that HPV acts as an oncogenic driver for this tumor [7]. Unlike this, the existence of HPV-negative cervical adenocarcinomas is accepted by most authors, estimated at approximately 15 to 38% [1,7,19].

Table 1 summarizes the prevalence rates of HPV-positivity in the various subtypes of cervical adenocarcinoma, classified according to the WHO (2020)/IECC (2018) [7,13,20,21].

**b.**  **False HPV-negative case**

Only some rare histotypes of cervical cancers are truly HPV negative, and a specific rate of false negative results at the HPV test must be considered. Thus, this may lead to an overestimation of the incidence of HPV-negative cancer in the literature [13,22,23]. 

Possible causes of false negative HPV tests are:

- *Loss of targeted HPV DNA fragment*

The HPV L1 fragment is usually well-conserved in most HPV genotypes and therefore is used as a target in many HPV tests. Integrating the viral DNA within the DNA host can destroy L1, L2, E1, and E2 fragments, resulting in a false negative HPV test. On the contrary, the expression of the oncogenes E6 and E7 is always present [5]. Tjalma et al. found that the HPV L1 test can miss 8.3% of HPV16 and 27.9% of HPV18 infections, compared with the HPV test covering E6 and E7 regions [24]. 

Another study showed that during the development of cervical cancer, there might be an inactivation of HPV, which no longer expresses the oncogenes E6 and E7 [25]. Therefore, it is desirable that cervical cancer screening should be based on tests targeting both the early (E) and the late (L) genes [24].

- *Low viral load in latent HPV infection*

Natural HPV infection has a latent period, during which the immune system fights viral replication and silences oncogenes. Latent infections have a low incidence of tumorigenesis. However, nearly 0.05% of HPV-negative cases can evolve into CIN 3 or cervical cancer in 3–5 years [26]. In these patients, the viral load is too low to be detected by the HPV test, with a higher risk of false negativity. Moreover, it must be considered that the glandular type epithelium does not support HPV viral infection. Therefore, in adenocarcinomas, HPV-DNA is often detected in the form of genomic integration. Summarizing, especially in adenocarcinomas, the use of sensitive HPV DNA detection tests is particularly essential [27].

- *Cervical cancer caused by low-risk HPV-genotype*

Most of the HPV tests currently in use target high oncogenic risk genotypes of HPV (HPV16 and HPV18), while they cannot reveal the presence of a low-risk HPV infection [27]. However, low oncogenic risk HPV (6, 11, 42, 44, 70) can cause cervical cancer [28,29]. According to Petry et al., these genotypes are detected in approximately 1–2% of primary cervical cancers [15]. It is still unclear whether their presence within malignant tumors represents the oncogenic driver or whether it is multiple accidental infections. In cervical adenocarcinomas, multiple HPV infections are present in approximately 8% of cases. In most cases, co-infections are represented by HPV genotypes 16 and 18 [30]. Multiple HPV infections in adenocarcinomas appear slightly lower than in the squamous counterpart. However, multiple HPV infections do not seem to influence the cancer process [31].

- *False negative HPV-test (incorrect sampling/pre-analytical errors)*

There are several HPV tests available in the market, each with different sensitivities and specificities. Nucleic acid signal amplification methods include polymerase chain reaction (PCR; e.g., Cobas^TM^ HPV and BD’s Onclarity^TM^ HPV) and transcription-mediated amplification (e.g., APTIMA^TM^ HPV).

Non-nucleic acid amplification methods are: hybridization capture (e.g., HC2^TM^) and invader chemistry (e.g., Cervista^TM^ HPV). Moreover, pathologists can use HPV hybridization in situ in formalin-fixed paraffin-embedded sections. HPV-tests currently validated by the Food and Drug Administration (FDA) for cervical cancer screening are: Hybrid Capture 2^TM^ (hybridization capture), Cervista^TM^ (invader chemistry), Cobas^TM^ (PCR), Onclarity^TM^ (PCR), APTIMA^TM^ (TMA) [5].

The most widespread cause of false negative HPV tests is the significant difference in the sampling procedures used by clinicians. Factors that can lead to false negatives are the following: sampling scanty cellular material from necrotic or inflamed areas, the presence of blood or lubricant in the sample, the time elapsed between excision and fixation, and degradation of viral DNA/RNA, especially in formalin-fixed and paraffin-embedded specimens [4,5].

A retrospective study showed that specimens from older patients and longer stored had a lower HPV positivity rate. In particular, storage time significantly influences the HPV positivity of adenocarcinomas more than squamous carcinomas [13].

**c.**  **Incorrect classification of a non-cervical cancer**

Histotypes of non-primary cervical cancers that may be misinterpreted are endometrial cancer extending to the cervix and metastases of HPV-negative tumors from other sites. A 2017 retrospective study reviewed cases of HPV-negative cervical cancer and found that more than two-thirds did not originate primarily from the cervix [15]. Therefore, endometrial carcinoma and HPV-negative cervical adenocarcinomas share a similar morphology, and tumor/stroma immunostaining is often required for differential diagnosis. Diagnosis of HPV-negative cervical adenocarcinoma is based on a combination of histology characteristics (for example, CD10 and CEA negativity) of the tumor and on the patient’s age (patients are usually younger than those with endometrial cancer) [7]. 

The tumors most frequently metastasize here are, in order of frequency: ovaries, colorectal, stomach, breast, kidney, and lungs [32]. Although it is not usually a site of metastasis, some studies indicate that 3.7% of female genital tract cancers extend to the cervix.

## 3. Classification of Endocervical Adenocarcinoma

The 2014 WHO Classification of cervical tumors distinguished ECA based exclusively on morphological and architectural features and the presence of intracytoplasmatic mucin [33]. This system did not account for clinical characteristics and was found to be poorly reproducible [34].

The newest IECC of 2018 attempted to categorize ECA by etiology (HPV status) and morphology with molecular and outcome evidence to give practitioners a more relevant clinical categorization [33,34,35,36].

The IECC was the result of a multi-institutional study that examined 409 cases of ECA from various countries (USA, Mexico, Romania, Japan, and Israel): it aimed to classify ECA based on HPV association. Then, their morphology was described and correlated with patients’ outcomes [33]. 

The first stratification of tumors was done by searching HPV-associated features: easily identifiable luminal or “floating” mitoses and/or apoptotic bodies and scanning magnification [33]. If they were not easily recognizable, visible only on high power, or not visible at all (no or limited HPV-associated features), the tumor has been considered non-HPV-related [33]. After this first subdivision, the ECA was further classified, considering the cytoplasmatic features to keep continuity with traditional classifications as delineated by 2014 WHO [33,36].

The last process includes IHC—p16, p53, vimentin, progesterone receptor PR- and RISH for HPV to validate this classification scheme. The method was concordant with IHC and RISH results: usual ECA shows 90% of p16 positivity and 95% of HPV-RISH; only 3% of NHPVA tumors showed HPV-RISH expression [33,36]. 

Other differences between HPVA and NHPVA were found in clinical aspects: NHPVA tumors were more extensive, occurred in older patients, and presented at a higher stage than HPV ECAs [4,5,33]. Subsequent studies have confirmed that IECC, compared with the WHO 2014 system, is more straightforward, practical, and reproducible, with a higher inter-observer agreement among pathologists [33,34]. Moreover, this new classification system is easy to perform in any laboratory since it only needs H&E (hematoxylin-eosin) staining without sophisticated additional tests [37]. 

The IECC 2018 created the basis for the latest WHO 2020 classification, which, for the first time, divides cervical epithelial tumors (including ECAs) and their precursor into different types based on HPV association. A recent workshop dedicated to ECAs, organized by the ISGyP in 2020, recommended categorizing ECAs according to the WHO 2020 classification system, which incorporates the IECC system [37].

The tumor types classified by the WHO 2014 and IECC 2018/WHO 2020 systems are reported in Table 2 [36,38,39].

## 4. Clinical Features of Human Papillomavirus-Negative Adenocarcinoma

### 4.1. Gastric-Type Adenocarcinoma (GCA)

**HPV.** Previous studies using PCR and in situ hybridization demonstrated that GCA is an HPV-negative tumor [40,41].

**Epidemiology.** GCA, described for the first time in the 1990s, is the most common type of NHPVA [42]. It is also the second most frequent histotype of cervical adenocarcinoma, accounting for approximately 10% of all ECA worldwide. It is frequent in Japan and reaches over 20% of all cervical adenocarcinomas [36,41]. The true incidence is still unknown because before the WHO 2020 classification, it was part of a spectrum of lesions ranging from MDA to undifferentiated adenocarcinoma, or it was confused with other histotypes (usual type, intestinal or clear cells) [43]. 

The mean age of the patients at the time of diagnosis is 52 years (ranging from 23–81), similar to the usual-type-adenocarcinoma [44]. GCA and its well-differentiated form of MDA often develop in patients with Peutz-Jeghers disease, a rare autosomal dominant syndrome associated with multiple gastrointestinal hamartomatous and mucocutaneous pigmentations [45]. 

**Symptoms and signs.** In contrast to usual-type ECA, GCA develops mainly in the upper part of the endocervix. The most reported symptoms are blood loss and intense watery discharge. Because of the highly infiltrative growth pattern, the most distinctive sign is an enlarged cervix without a well-defined mass [46].

**Pap test.** Pap-test can result as normal in 30–50% of cases, while other 50–70% of patients receive an abnormal cytology result not specific to mucinous adenocarcinoma [47,48].

**Colposcopy.** The most frequent localization of the GCA is the upper part of the cervix, so during colposcopy, the transformation zone can appear normal. However, the cervix has an enlarged appearance, with a stiffer consistency, without a well-defined mass [47,49].

**Radiodiagnostics.** At the ultrasound exam, GCA appears as a multilocular cystic mass and can present hypervascularization with Power Doppler. On MRI, the cervix seems enlarged and has a heterogeneous pattern, with T2 hyperintensity and enhancement [47,50]. 

**Precursors.** Several cases of GCA appear to originate from precursor lesions, such as simple gastric hyperplasia and LEGH, a benign form occasionally associated with Peutz-Jeghers syndrome [51]. LEGH is often found in the upper cervix in the context of GCA, and histological examination shows a gastric-markers lobular architecture. Moreover, the average age of patients with LEGH is 45–49 years, similar to GCA [42,51]. These characteristics and the fact that it shares the same genetic mutations of the GCA (KRAS, STK11, TP53, and others) make it a probable benign precursor of GCA [48,52,53,54].

There are also atypical forms of LEGH, variants of the GAIS, which show an atypical histological architecture (loss of polarity, papillary arrangements, enlargement of cell nuclei, evident nucleoli, etc.,) without invasion of the stroma [55].

GAIS and GCA frequently occur together and share the same genetic profile (e.g., acquisition of chromosome 3p and loss of 1p), indicating that LEGH, GAIS, and GCA are different shades of HPV-independent cervical adenocarcinoma with gastric differentiation [56].

**Prognosis.** GCA shows aggressive behavior and is associated with a lower survival rate than the usual-type adenocarcinoma, even in stage I, because of its higher probability of invasion, metastasis, and chemoresistance. The 5-year overall survival rate reported in the literature is 30% to 43% for gastric-type NHPVA, compared to 74–91% for usual-type adenocarcinoma [49,57].

GCA shows higher rates of lymphovascular invasion, depth and horizontal invasion, parametrial and vaginal extension, regional and distant lymph node metastasis, and ascitic fluid than the usual endocervical type adenocarcinoma [47,48,57,58]. Moreover, it usually presents in an advanced stage of the disease, probably because of its negativity to HPV-test and because the cytology has a low sensibility for MDA since cytologic aspects of GCA are a recent acquisition [59].

At the time of diagnosis, 40–100% of patients are stages II-IV, while 75% can present metastasis to distant organs (especially lung, ovary, liver, colon, and bone) [49,57,58].

As the new guidelines indicate the HPV test is the best screening test for cervical cancer, Omori et al. proposed adding a further molecular test for gastric mucin to the HPV test. Using a monoclonal antibody against gastric mucin showed an excellent positive predictive value for gastric-type benign and malignant lesions [60].

### 4.2. Clear Cell Adenocarcinoma (CCC)

**HPV.** CCC seems to be associated with HPV infection only in 25–30.4% of cases [5]. 

**Epidemiology.** Clear cell carcinoma represents 2–7% of cervical adenocarcinomas [7]. Although its etiology is unknown, CCC has been linked to in-utero exposure to synthetic oral estrogen, DES, widely used in several countries between 1948 and 1970 to prevent miscarriage and preterm birth [61]. In the DES-exposed group, the peak incidence is reached at 19 years and remains high throughout life. The non-DES-exposed group has no peak age because the incidence ranges from pediatric to postmenopausal [4]. In addition, some cases of CCC in non-DES-and-HPV-exposed children and young women have been reported [62,63]. In DES-exposed patients, the most frequently involved zones are the ectocervix and the anterior upper part of the vagina. Instead, in women with non in utero-DES-exposure, CCC mainly develops in the endocervix [7,33]. In utero exposure to DES can cause both CCC and genito-urinary malformations. Several cases of CCC arise in women with double uteri and vagina with unilateral renal agenesis, atresic hemicervix, and ipsilateral renal agenesis or bicornuate uterus [64]. 

**Symptoms and signs.** CCC can present with cervical ulceration that causes abnormal vaginal bleeding (postcoital bleeding, intermenstrual bleeding), usually refractory to hormonal therapy [65]. At the gynecological examination, an abnormality in the consistency of the cervix can sometimes be perceived as “fullness” [66].

**Pap test.** Cytology is poorly sensitive in diagnosing CCC, and previous clinical research reported that only 18% of patients with CCC had an abnormal Pap test [67,68]. 

**Radiodiagnostics.** CCC at the MRI shows hypointensity on T1-weighted images, hyperintensity on T2-weighted images, and heterogeneous enhancement [66]. 

**Precursors.** Although the pathogenesis of CCC is not still understood, its genetic profile suggests that, in non-DES-exposed cases, CCC can arise from areas of ectocervical adenosis or cervical endometriosis [69]. Moreover, Talia et al. assumed that even the tubo-endometrial metaplasia adjacent to the cervix could be a precursor of CCC [70]. 

**Prognosis.** Previous literature about CCC prognosis is controversial: some authors report the exact outcomes of usual-type adenocarcinoma, whereas others notice more aggressive behavior [66]. 

Interestingly, Wang et al. reported a better prognosis in DES-exposed CCC patients than those with spontaneous CCC [71]. CCC is more aggressive than squamous cell carcinomas in recurrence and metastasis [72,73]. Local recurrence may occur more frequently than disease dissemination to other organs. Metastasis occurs in about 18% of I-stage diseases and nearly 50% of II-stage tumors. The lungs, liver, and bones are the most common extra-pelvic sites [74]. Most recurrences of CCC of the uterine cervix get diagnosed within three years after primary tumor treatment, whereas late recurrences are less frequent [63]. 

The survival rate of patients with FIGO stages I/II ranges from 81.5% to 91%, and 57% of patients are still alive after a 10-year follow-up [67]. 

### 4.3. Mesonephric Adenocarcinoma

**HPV.** Mesonephric adenocarcinoma seems to be not associated with HPV infection [75]. 

**Epidemiology.** The average age of patients is 52, ranging from 35 to 72 years. Its prevalence has a homogeneous distribution in the various periods from the 3rd to the 6th decade, and there is no age in which it has a peak incidence [76]. 

**Symptoms and signs.** It usually develops in the lateral-to-posterior part of the cervix. It can rarely involve the entire cervix without forming a well-defined mass. Its growth pattern can be invasive, bulky, or exophytic [75].

**Pap test.** Pap tests performed during routine screening can detect cytological abnormalities, but such detection occurs less frequently than squamous cervical cancer of the cervix. The diagnosis is often obtained through cervical biopsy, curettage, or hysteroscopy [77,78]. 

**Precursors.** Mesonephric adenocarcinoma is presumed to originate from normal or hyperplastic mesonephric duct remnants located in 22% of adult women in the lateral part of the cervix [76,77].

**Prognosis.** About 70% of the patients are diagnosed at stage IB. The recurrence rate is 32%, with a mean recurrence interval of 24 months. This tumor has an aggressive behavior and can metastasize early to distant organs, so the overall prognosis remains less favorable compared to other cervical adenocarcinomas. The average survival time is about 50 months [76,78]. 

### 4.4. Endometrioid Adenocarcinoma (ENAC)

**HPV.** HPV is identified in 0–27% of cases of ENAC. In particular, HPV is detected in 100% of ENAC arising from the squamocolumnar junction [79,80,81]. In contrast, ENACs that develop from the upper endocervix and lower uterine segments are HPV-independent [1,13,82].

**Epidemiology.** This tumor is sporadic and accounts for less than 5% of cases of cervical adenocarcinoma. The mean age of incidence of ENAC ranges from 43 to 50 years [79,83]. 

It is estimated that using the new WHO 2020 guidelines for diagnosis, only 1.1% of endometrioid adenocarcinomas previously identified using the WHO 2014 classification would be confirmed [36]. 

Given its low incidence, more common pictures such as endometrioid adenocarcinoma of the endometrium extending into the cervix or unusual presentations of usual-type adenocarcinoma should be suspected before diagnosing ENAC [4]. 

**Precursors.** Endometrioid cancer reportedly develops from cervical endometriosis. Some authors suspected ENAC could arise from a malignant transformation of endometriotic areas [84,85]. 

**Prognosis.** Because of its rarity, data about ENAC prognosis are still limited. A recent study compared usual-type adenocarcinoma with endometrioid adenocarcinoma in terms of overall and disease-specific survival and found no statistically significant difference between them. The 5-years overall survival for ECA was 75.6% [86].

## 5. Molecular Characterization of Non-HPV Associated Adenocarcinoma

### 5.1. Gastric-Type Adenocarcinoma 

The most common gastric-type NHPVA genetic alterations reported in the literature are TP53, STK11, KRAS, ARID1A, BRCA2, CDKN2A, and others (Table 3) [8,44,48,54,87,88].

This package of mutations is similar to the genomic profile of pancreaticobiliary adenocarcinoma, which resembles endocervical adenocarcinoma from a morphological and molecular point of view [33].

Garg et al. using NGS, investigated 161 cancer-driver genes and copy-number variations, gene fusions, and insertions/deletions in 14 gastric-type endocervical adenocarcinomas. They detected a group of mutations involved in DNA repair mechanisms, cell cycle, Fanconi anemia, and PI3K-AKT signaling [48].

Other studies assessed that TP53, STK11, CDKN2A, ATM, and NTRK3 mutations are more frequent in gastric-type endocervical adenocarcinoma than the usual type adenocarcinoma, while PIK3CA seems to be less frequent [44,54]. 

In addition to detecting mutations of TP53 (53%), STK11 (33%), and CDKN2A (27%), Lu et al. showed amplification of the ERBB2 gene in 13% of patients [87]. 

**TP53:** TP53 is a gene activated in response to several forms of cellular stress (i.e., DNA damage, oncogene expression, and hypoxia) and exerts multiple antiproliferative functions. Its transcription factor p53 stimulates the expression of p21, an inhibitor of mitosis transitions. In some cases, the activation of p53 leads to apoptosis by activating the Bax protein and stimulates the expression of genes that prevent blood vessel formation, a fundamental process in tumor growth [89].

Loss of p53 occurs due to E6 protein in usual-type HPV-related adenocarcinomas, while in GCA, which are entirely HPV-independent, it occurs due to somatic mutation of the TP53 gene.

An ever-increasing body of literature demonstrated that TP53 is the most frequently mutated gene in GCA (see Table 3) [48]. Interestingly, the frequency of TP53 mutation seems to be higher in patients with HPV-independent cervical adenocarcinoma than in HPV-positive patients [90]. 

Some authors demonstrated that p53 overexpression is more frequent in GCA than in other cervical histotypes and represents an independent prognostic factor for poor disease-specific survival and risk of tumor recurrence [91].

**STK11:** the serine/threonine kinase 11 (STK11), also known as liver kinase B1 (LKB1), is a tumor suppressor gene. Its mutation in the inactivating sense was found in Peutz-Jeghers syndrome and several sporadic tumors [92]. In particular, Peutz-Jeghers syndrome is caused by a germline mutation of STK11, with autosomal dominant inheritance, and it is associated with the development of MDA, a well-differentiated form of cervical adenocarcinoma. Moreover, Kuragaki et al. found sporadic STK11 mutations in 55% of 11 MDA [45,93]. Now that it is accepted that MDA is part of the morphological spectrum of gastric-type ECA, it has been understood that the STK11 mutation, both sporadic and germline, belongs to gastric-type adenocarcinoma [94].

Understanding the mechanism of action of STK11 is crucial since it seems to be an independent adverse prognostic factor associated with increased mortality [90]. In HPV-associated cervical cancer, Zeng et al. demonstrated that the loss of copies of STK11 amplifies the HPV16 E6/E7 oncogenic potential and leads to increased cell growth and metastatic invasion [92].

In gastric-type adenocarcinoma, STK11 mutation is associated with significantly lower survival and more extensive lymphovascular invasion regardless of the FIGO stage [87].

While its involvement in HPV-associated cancer is partially elucidated, the significance of the STK11 mutation in NHPVA adenocarcinoma has yet to be investigated. Its role in HPV-independent oncogenesis must be further understood for target therapies to be used in the future.

**APOBEC3.** APOBEC3 is associated with some drivers mutation and is thought to play an important role in cervical cancer pathogenesis. 

A recent study among the Chinese population found a gain of somatic copy number of APOBEC3B in 20% of 25 GCA cases, while it was absent in usual-type adenocarcinoma. Interestingly, a high positivity to APOBEC3B was associated with a favorable prognosis. Thus, in the future APOBEC3B could become a potential prognostic value of lower relapse risk of GCA, independently from the tumor stage [95].

**ERBB2.** The ERBB2 gene encodes the HER2 protein, the human epidermal growth factor receptor 2. Amplification of the ERBB2 gene is associated with breast, ovary, gastric, colorectal, and uterus carcinoma. 

A recent study analyzed 209 cases of adenocarcinoma, including 159 HPV-related and 50 instances of NHPVA. While in 10% of mucinous-type adenocarcinoma, HER2 amplification was detected, in the GCA patients, the frequency of HER2 amplification was 14.7%, and it was the highest value of all histotypes. Moreover, HER2 was associated with increased tumor stage (FIGO III/IV), perineural involvement, lymphovascular invasion, and ovarian spread.

If, on the one hand finding HER2 amplification in a patient with adenocarcinoma worsens the prognosis, from another point of view, it makes her susceptible to Trastuzumab therapy.

Gene amplification’s predictive value needs further investigation. Other studies are necessary to decide the best technique (immunohistochemistry or fluorescence in situ hybridization) to search for HER amplification on histological examination [91].

### 5.2. Clear Cell Adenocarcinoma (CCC)

CCC is a rare variant of cervical adenocarcinoma. Thus, data about its molecular profile remain limited. Microsatellite instability was found in 100% of DES-exposed women, but it was present in only 50% of non-DES-exposed cases [96].

Recently, Lee et al. found in CCC DES-exposed women the POLE gene mutation associated with increased tumor-infiltrating lymphocytes and high PD-L1 expression [97]. 

The involvement of mismatch repair genes is unclear: Mills et al. found a case of CCC with MMR deficiency but without MMR proteins. At the same time, Nakamura et al. reported a patient with Lynch Syndrome who developed a synchronous CCC with loss of MSH2 and MSH6 [98,99].

It is also hypothesized that the PI3K-AKT pathway is involved in the pathogenesis of CCC. Ueno et al. found p-AKT and p-mTOR positivity in 50% of cases; elderly patients had a loss of PTEN in 50% of cases, an increase in EGFR in 75%, and an amplification of HER2 in 50% of cases [21].

### 5.3. Mesonephric Adenocarcinoma 

Previous studies suggest that the molecular profile of mesonephric carcinoma is characterized by the KRAS mutation, which, unlike other cervical carcinomas, is present in 81% of cases, often associated with the activating mutation of NRSA [100]. 

This histotype also shows mutations of ARID1A/B and SMARCA4 (in 62% of cases) and BCOR/BCORL1 (in 33% of cases). No alterations in PTEN, ARID1A, TP53, or microsatellite instabilities were found [100]. 

Two recent case reports found the FGFR2 mutation in two cases with mesonephric adenocarcinoma. In particular, Devarashetty et al., after surgical treatment, offered the patient a targeted therapy of tyrosine kinase inhibitors and immunotherapy, with an excellent response. Thus, FGFR2 mutation is one of the possible genetic abnormalities in this histological type and needs to be searched to offer the patient a targeted treatment [77].

At the chromosomal abnormalities analysis, 71% of tumors exhibit 1q gain, often accompanied by 1p loss. In addition, 57% of these tumors harbor chromosome 10 gain, frequently associated with chromosome 12 gain [7,101]. 

Even though mesonephric adenocarcinoma is thought to originate from hyperplastic mesonephric remnants, the pathogenesis toward malignity is unclear. According to Kim et al., mesonephric atypical hyperplasia could be a pre-invasive lesion that evolves into mesonephric carcinoma through the gain of KRAS and chromosome 1 mutation [102].

### 5.4. Endometrioid Adenocarcinoma 

The molecular characteristics of endometrioid carcinoma are still unknown. In 2020, Jenkins et al. analyzed the genome of eight cases of HPV-negative endometrioid adenocarcinoma and detected several somatic genetic mutations: PIK3CA (50%), PTEN (50%), CTNNB1, FBXW7, KRAS, AKT1, MSI-H. In the future, it will be helpful to identify genetic similarities with endometriosis-related carcinomas or molecular peculiarities that may help in the differential diagnosis of endometrial cancer that extends to the cervix [8].

### 5.5. PD1 and PDL1 Expression in NHPVA

PDL1 and PD1 are proteins expressed by immunity cells (lymphocytes, macrophages, antigen-presenting cells, APC, and thymic epithelial cells). The interaction between PD1 and PDL1 promotes T-cell apoptosis and functional exhaustion [103]. The interaction between PD1 and PDL1 also prevents the activation of the cells’ immune response and their cytotoxic response against tumor cells [104,105].

The block of the PD1-PDL1 axis by anti-PD1 or anti-PDL1 antibodies has been proven to be a successful strategy for interrupting tumor immune tolerance and enhancing an antineoplastic immune response in several tumors [106]. Studies about PDL1 expression in NHPVA are limited. Recently, Song and Chen investigated PDL1 expression among different histotypes of NHPVA and its correlation with prognosis, progression-free survival, and overall survival [107,108].

Positivity for PDL1 was evaluated using a combined positive score (CPS) and tumor proportion score (TPS). CPS is defined as the number of PDL1 stained cells (tumor cells, lymphocytes, and macrophages) divided by the total number of viable tumor cells and then multiplied by 100. TPS is the percentage of viable tumor cells showing partial or complete membrane staining adenocarcinoma positive or PDL1 expression [107,108].

Song et al. observed no statistical difference in PDL1 expression between the different histological types of adenocarcinomas when evaluating PDL1 positivity through CPS. However, PDL1 showed high positivity in clear cell carcinomas compared to gastric-type adenocarcinoma. This study showed a higher association between PDL1 positivity and CCC when TPS defined PDL1 positivity (58.3% PDL1 positivity vs. 14.3% positivity for gastric type adenocarcinoma and 40% positivity for endometrioid adenocarcinoma). PDL1 was not expressed in mesonephric carcinoma cases [107].

PDL1 expression was significantly associated with a high tumor-infiltrating lymphocyte percentage and with worse progressive free survival and overall survival rate [107].

Chen et al. studied PDL1 expression in NHPVA, focusing on gastric-type adenocarcinoma, and compared it with HPVA [108]. They observed no statistically significant difference in the expression of PDL1 between HPVA and HPV-independent lesions. However, they also observed that PDL1 expression correlates with progression-free survival and survival in patients with HPV-independent gastric-type adenocarcinoma [108].

**Figure 1:**
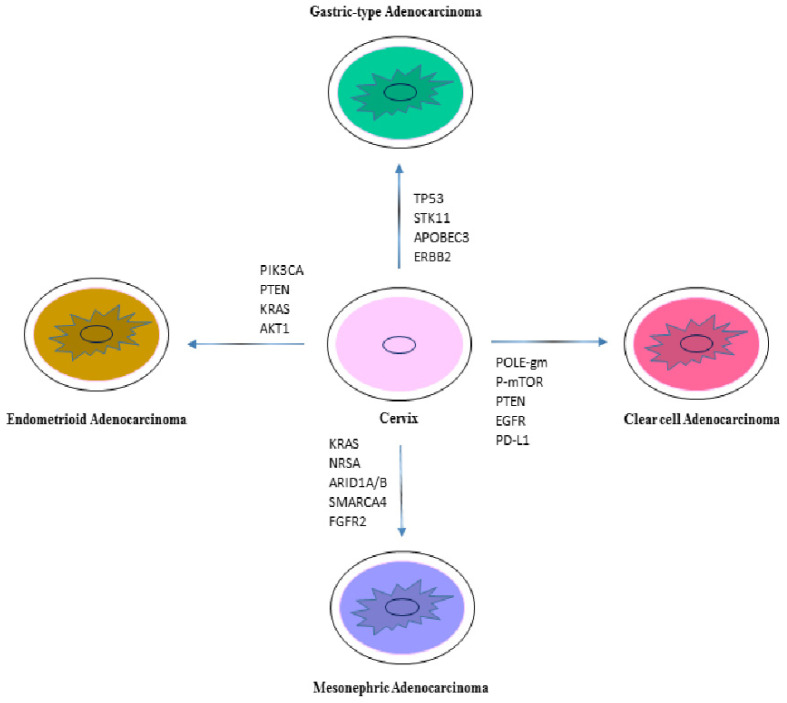
Main gene mutations in different HPV-negative cervical adenocarcinomas.

A scheme of the significant gene mutations in HPV-negative adenocarcinomas is shown in Figure 1.

## 6. Treatment of Human Papillomavirus-Negative Cervical Cancer 

### 6.1. Staging

In patients with HPV-unrelated cervical adenocarcinoma, treatment planning should be established on a multidisciplinary basis (e.g., a tumor board meeting). The choice of options must be based on the accurate knowledge of prognostic and predictive factors for an oncological outcome, quality of life, and morbidity [109]. A team of specialists dedicated to oncological gynecology should carry out appropriate counseling.

According to The European Society of Gynaecological Oncology (ESGO)/European Society for Radiotherapy and Oncology/European Society of Pathology Guidelines for the Management of Patients with Cervical Cancer [109] and the National Comprehensive Cancer Network (NCCN) guidelines for Cervical Cancer [110], staging should be performed according to the TNM classification, and clinical staging (FIGO) should be recorded.

It is recommended to record [109]:

1. *TNM and FIGO stage, including maximum tumor size and detailed description of extracervical tumor extension and nodal involvement.*
2. *Pathological tumor type.*
3. *Depth of cervical stromal invasion and a minimum thickness of uninvolved cervical stroma.*
4. *Presence (or absence) of lymphovascular space involvement (LVSI).*
5. *Presence or absence of distant metastases.*

Diagnostic workup includes: (1) pelvic examination and biopsy (with or without colposcopy) are mandatory; (2) pelvic MRI is necessary to assess pelvic tumor extent; (3) endovaginal/transrectal ultrasound is an option if performed by an expert sonographer; (4) cystoscopy or rectoscopy may be considered to provide a biopsy if suspicious lesions in the urinary bladder or rectum are documented on MRI or ultrasound. 

Diagnosis of a T1a tumor must be based on a conization specimen examined by an expert pathologist. The depth of the cervical conization may be tailored to the lesion’s size, location, and histotype (if known). In a patient with suspicion of invasive adenocarcinoma versus adenocarcinoma in situ, the cone biopsy would be designed as a cylindrical cone extending to the internal os to exclude possible invasion in the endocervical canal. Length of the cold cone of at least 10 mm is preferred and can be increased to 18–20 mm in patients who have completed childbearing [111,112].

Distant diagnostic workup, according to ESGO guidelines [109], includes:

1. In the early stage (T1a, T1b1, T2a1), surgical/pathological staging of pelvic lymph nodes is the gold standard to assess the prognosis and guide treatment (except for T1a1 and no LVSI).
2. In locally advanced cervical cancer T1b2 and higher (except T2a1) or early stage disease with suspicious lymph nodes on imaging, positron emission tomography-computed tomography (PET-CT) or chest/abdomen computed tomography (CT) is recommended for assessment of nodal and distant disease.
3. Paraaortic lymph node dissection, at least up to the inferior mesenteric artery, may be considered in locally advanced cervical cancer with negative paraaortic lymph nodes on imaging for staging purposes.

NCCN guidelines differentiate diagnostic workups based on the desire for fertility-sparing and the clinical stage of the disease [110]. 

### 6.2. Mangement of Stage T1a

Microinvasive disease (stage Ia1) with no LVSI has less than a 1% risk of lymphatic metastasis. A patient with squamous cell carcinoma or usual-type (HPV-related) carcinoma may be managed conservatively. This is important for preserving fertility with cone biopsy with negative margins or with simple extrafascial hysterectomy when fertility preservation is not required. 

In patients with non-HPV associated subtypes of endocervical adenocarcinoma, treatment of stage T1a (T1a1 and T1a2) differ from other histotypes [109,110]: according to the ESGO guidelines, in a patient with rare histological subtypes of cervical cancer including non-HPV-related adenocarcinomas (except for adenoid basal carcinoma), and neuroendocrine carcinomas, fertility-sparing treatment should not be recommended [109]. NCCN guidelines do not allow conservative treatment in the event of gastric type adenocarcinoma, or adenoma malignum (minimal deviation adenocarcinoma), or in case of small cell neuroendocrine histology because of their high-risk nature and a lack of data [110]. 

### 6.3. Management of Other Stages (T1b/TIV)

Surgery is typically reserved for early stage diseases, such as stage Ia, Ib1, Ib2, and IIa1 [113]. Concurrent chemoradiation is generally the primary treatment for stages IB3 to IVA disease, based on five randomized clinical trials [114,115] and for patients who are not candidates for hysterectomy. Although few studies have assessed treatment for cervical adenocarcinomas, they are typically treated similarly to squamous cell carcinomas [109,110,116,117,118]. Ovarian preservation cannot be proposed in the case of HPV-unrelated cervical adenocarcinoma.

Adjuvant treatment is indicated after radical hysterectomy, depending on surgical findings and disease stage. Adenocarcinoma (in particular, adenocarcinoma non-HPV related) is considered an additional risk factor [119,120].

## Figures and Tables

**Table 1:** HPV-positivity rate according to histologic subtypes.

	HPVA	NHPVA	*Ref.*
Adenocarcinoma Histology	HPV Positivity Rate (%)		
Usual type	72–100	-	*[7,13,20]*
Mucinous	83–100	-	*[7]*
Gastric type	-	0	*[20]*
Clear cells type	-	0–28	*[13,20,21]*
Mesonephric type	-	0	*[7]*
Endometrioid type	-	0–27	*[7,13,20]*

**Table 2:** Classifications of adenocarcinoma of the uterine cervix.

WHO 2014	IECC 2018/WHO 2020	
	HPV-Associated (HPVA)	Non-HPV-Associated (NHPVA)
Usual type	Usual type	Gastric type
Mucinous carcinoma, NOS	Villoglandular	Clear cells
Gastric type	Mucinous, NOS	Mesonephric
Intestinal type	Mucinous, intestinal	Endometrioid
Signet ring cell	Invasive stratified mucin-producing	
Villoglandular	Micropapillary	
Endometrioid	Serous’-like	
Clear cells		
Serous		
Mesonephric		

**Table 3:** Genetic mutations and gene amplification in gastric-type adenocarcinoma.

Genetic Mutation	Sample Size (Number of Patients)	Cases with Genetic Mutation (%)	*Ref.*
AKT1	11	33	*[54]*
ARID1A	14	29	*[48]*
ARID1A	15	20	*[87]*
ATM	11	18	*[54]*
BRCA2	14	21	*[48]*
BRCA2	21	10	*[88]*
CDKN2A	3	67	*[8]*
CDKN2A	14	36	*[48]*
CDKN2A	15	27	*[87]*
CDKN2A	68	18	*[44]*
ELF	11	18	*[54]*
ERBB2	68	9	*[44]*
ERBB3	21	10	*[88]*
ERBB3	68	10	*[44]*
FGFR4	21	14	*[88]*
GNAS	21	10	*[88]*
GNAS	68	9	*[44]*
HLA-B	21	19	*[88]*
KMT2D	11	18	*[54]*
KRAS	3	33	*[8]*
KRAS	11	36	*[54]*
KRAS	68	17	*[44]*
MSH2	14	21	*[48]*
MSH6	14	43	*[48]*
NTRK3	11	18	*[54]*
PIK3CA	11	18	*[54]*
PIK3CA	68	7	*[44]*
POLE	14	36	*[48]*
PTEN	15	20	*[87]*
PTPRS	21	19	*[88]*
SLX4	14	36	*[48]*
SLX4	14	36	*[48]*
SLX4	21	10	*[88]*
SMAD4	68	9	*[44]*
STK11	3	33	*[8]*
STK11	14	29	*[48]*
STK11	15	33	*[88]*
STK11	19	21	*[88]*
STK11	68	10	*[44]*
TP53	3	67	*[8]*
TP53	11	46	*[54]*
TP53	14	50	*[48]*
TP53	15	53	*[87]*
TP53	21	52,4	*[88]*
TP53	68	41	*[44]*
CDK12	15	7	*[87]*
ERBB2	15	13	*[87]*
MDM2	14	14	*[48]*
MECOM	15	7	*[87]*

## Abbreviations

AKT1	Serine-threonine protein kinase AKT1
APOBEC3	Apolipoprotein B mRNA editing enzyme, catalytic subunit 3
ARID1A	AT-rich interactive domain-containing protein 1A
ATM	Ataxia telangiectasia mutated
BCOR	BCL6 corepressor
BRCA2	Breast cancer gene 2
CCC	Clear cell adenocarcinoma
CDK12	Cyclin dependent kinase 12
CDKN2A	Cyclin dependent kinase inhibitor 2
CIN	Cervical intraepithelial neoplasia
CTNNB1	Catenin Beta 1
DES	Diethylstilbestrol
DNA	Deoxyribonucleic acid
ECA	endocervical adenocarcinoma
EGFR	Epidermal growth factor receptor
ELF	Ets domain transcription factor
ENAC	Endometrioid adenocarcinoma
ERB2	Erb-B2 receptor tyrosine kinase 2
FBXW7	F-box and WD repeat domain containing 7
FDA	Food and Drug Administration
FGFR2	fibroblast growth factor receptor 2
FGFR4	Fibroblast growth factor receptor 4
GAIS	Gastric-type adenocarcinoma in situ
GCA	Gastric-type adenocarcinoma
GNAS	Guanine nucleotide binding protein
H&E	hematoxylin eosin
HER2	Human epidermal growth factor receptor 2
HLA-B	Human leukocyte antigen-B
HPV	Human papillomavirus
IECC	International endocervical criteria and classification
IHC	immunohistochemistry
ISGyP	International Society of Gynecological Pathologists
KMT2D	Lysine methyltransferase 2D
KRAS	Kirsten rat sarcoma
LEGH	Lobular endocervical glandular hyperplasia
MDA	Minimal-deviation adenocarcinoma
MDM2	Mouse double minute 2
MECOM	MDS1 and EVI1 complex locus
MMA	DNA mismatch repair
MRI	Magnetic resonance imaging
MSH2	MutS homolog 2
MSH6	MutS homolog 6
MSI-H	microsatellite instability-high
NGS	Next generation sequencing
NHPVA	non-HPV adenocarcinoma
NTRK3	Neurotrophic receptor tyrosine kinase 3
p-mTOR	Phosphorylated mammalian target of rapamycin
PCR	Polymerase chain reaction
PD-L1	programmed cell death ligand 1
PI3K-AKT	Phosphatidylinositol-3-kinase
PIK3CA	Phosphatidylinositol-4,5-bisphosphate 3-kinase catalytic subunit alpha
POLE	polymerase epsilon
PTEN	Phosphatase and tensin homolog
PTPRS	Protein tyrosine phosphatase receptor type S
RISH	RNA in-situ hybridization
RNA	Ribonucleic acid
SCC	Squamous cell carcinoma
SLX4	Structure-specific endonuclease subunit
SMAD4	Mothers against decapentaplegic homolog 4
SMARCA4	SWI/SNF related, matrix associated, actin dependent regulator of chromatin, subfamily A, member 4
STK11	Serine/threonine kinase 11
TP53	Tumor protein p53
WHO	World Health Organization
